# Assessing sex differences in viral load suppression and reported deaths using routinely collected program data from PEPFAR-supported countries in sub-Saharan Africa

**DOI:** 10.1186/s12889-023-16453-6

**Published:** 2023-10-07

**Authors:** Danielle Fernandez, Hammad Ali, Sherri Pals, George Alemnji, Vamsi Vasireddy, George K. Siberry, PEPFAR MER Interagency Participants, Yolanda Rebello Cardoso, Caroll Vasquez, Emily Bruno, Apollinaire Kavungerwa, Franck Kavabushi, Aime Ndayizeye, Laura T. Eno, Zacheaus Zeh Akiy, Serge Clotaire Billong, Elie Mukinda, Solomon Ahmed, Daniel Fiseha, Zerihun Hika, Anthony Waruru, Fred Asiimwe, Misheck Luhanga, Faustin Matchere, Jordan McOwen, Gram Mutandi, Leonard Bikinesi, Laimi Ashipala, Ibrahim T. Dalhatu, Alex Bolo, Joel Sua Katoro, Mushubira M. Balinda, Matthew Rosenthal, Boniface Nguhuni, Alex S. Magesa, Ikwo Oboho, Catherine Godfrey

**Affiliations:** 1 Public Health Institute (PHI), CDC Global Health Fellowship Program, Atlanta, USA; 2 Division of Global HIV and Tuberculosis, Center for Global Health, U.S. Centers for Disease Control and Prevention (CDC), Atlanta, USA; 3 Walter Reed Army Institute of Research, U.S. Department of Defense (DOD), U.S. Embassy, Kampala, Uganda; 4 United States Agency for International Development (USAID), Washington, D.C USA; 5 Office of the Global AIDS Coordinator, U.S. Department of State, Washington, D.C USA

**Keywords:** Sex differences, Viral suppression, Interruption in treatment, Mortality, PEPFAR

## Abstract

**Background:**

In sub-Saharan Africa, more women than men access HIV testing and treatment and may have better viral load suppression (VLS). We utilized routinely reported aggregated HIV program data from 21 sub-Saharan African countries to examine sex differences in VLS and death rates within antiretroviral therapy (ART) programs supported by the United States President's Emergency Plan for AIDS Relief (PEPFAR).

**Methods:**

We included VLS and reported death data for persons aged 15 + years on ART from October–December 2020 disaggregated by sex and age for each subnational unit (SNU). We used linear mixed-model regression to estimate VLS proportion and negative binomial mixed-model regression to estimate the rates of death and death plus interruptions in treatment (IIT). All models were weighted for SNU-level ART population size and adjusted for sex, age, HIV/tuberculosis coinfection, country, and SNU; models for reported deaths and deaths plus IIT were also adjusted for SNU-level VLS.

**Results:**

Mean VLS proportion was higher among women than men (93.0% vs. 92.0%, *p*-value < 0.0001) and 50 + than 15–49 age group (93.7% vs. 91.2%, *p*-value < 0.0001). The mean rate of reported deaths was higher among men than women (2.37 vs. 1.51 per 1000 persons, *p*-value < 0.0001) and 50 + than 15–49 age group (2.39 vs. 1.50 per 1000, *p*-value < 0.0001); the mean rate of reported deaths plus IIT was higher among men (30.1 in men vs. 26.0 in women per 1000, *p*-value < 0.0001) and higher among 15–49 than 50 + age group (34.7 vs. 22.6 per 1000, *p*-value < 0.0001).

**Conclusions:**

The mean rate of reported deaths was higher among men in most models despite adjusting for VLS. Further exploration into differences in care-seeking behaviors; coverage of screening, prophylaxis, and/or treatment of opportunistic infections; and more extensive testing options for men to include CD4 is recommended.

## Background

With the global scale-up of antiretroviral therapy (ART), HIV outcomes have improved worldwide with declining HIV incidence and HIV-related mortality [1]. The latest Joint United Nations Programme on HIV/AIDS (UNAIDS) estimates indicate that global HIV incidence in adults 15 years and older decreased from 0.56 per 1000 persons in 2000 to 0.23 per 1000 persons in 2020 [2]. HIV mortality similarly declined with 580,000 deaths due to HIV/AIDS worldwide in 2020 compared to 1.2 million in 2000, a 52% decrease in two decades. However, disparities persist in the incidence, severity of HIV infection, and mortality in various geographic regions and among certain populations.

Sex differences in the biology and immunology of HIV have been well-documented. A review examining sex differences in plasma HIV RNA levels at similar infection points reported that 7 of 9 cross-sectional studies found lower RNA levels in women despite controlling for CD4 cell count and all longitudinal studies included in the review (n = 4) found similar results despite controlling for time since seroconversion [3]. HIV RNA levels measure viral load and CD4 count provides an indication of immune function in people living with HIV (PLHIV) and is one of the key determinants for determining need for opportunistic infection (OI) prophylaxis. However, multiple studies have found that, while women have lower viral load measurements in untreated and early HIV-1 infection, this biological benefit diminishes over time as disease progression is similar between the sexes [4–6]. Furthermore, findings from a 2016 clinic-based study conducted in Kenya, Mozambique, Rwanda, and Tanzania suggest that sex differences in all-cause mortality and loss to follow-up are observed among PLHIV across all age bands, and that women on ART appear to have disproportionate risk of death as they age, compared to men [7].

Advanced HIV disease (AHD) is defined as having CD4 count < 200 cells/mm^3^ or it can be staged clinically. A 2013 cohort study of PLHIV in Tanzania found that at initiation of treatment, women had less advanced HIV disease than men [33% versus (vs.) 47%] and higher median CD4 counts than men (149 cells/mm^3^ vs. 102 cells/ mm^3^). After 1 year of ART, a higher proportion of women had undetectable plasma viral load than men (69% vs. 45%), however, CD4 cell counts were comparable. No significant difference in the survival rate after 1 year of treatment between men and women was observed, suggesting that the response to treatment between the sexes is similar (relative hazard 1.02, 95% CI 0.75, 1.38) [8]. Data from a nationwide laboratory cohort in South Africa found that, from 2005 to 2011, the proportion of patients entering care with CD4 count < 200 cells/mm^3^ declined but remained stable from 2011 onward. Among patients entering care for the first time, 32.9% had AHD, 16.8% had very advanced HIV disease (defined in the study as CD4 < 100 cells/mm^3^), and men were nearly twice as likely as women to enter care already with very advanced HIV disease [9]. Over the last decade, the pattern of individuals presenting with advanced HIV has changed. A study in South Africa, assessing the burden of AHD over 10 years, found that ART-naïve patients initially constituted the majority of those with CD4 count < 50 cells/mm^3^; but a shift occurred over the decade, and the contribution of ART experienced patients to this group increased from 14.3% to 56.7%, becoming the largest proportion of those with AHD. In 2016, the final year of the study, in patients with CD4 count < 50 cells/mm^3^, 51.8% were ART experienced, of whom 76% were confirmed to not be on ART at the time, and more than half were men [10].

In sub-Saharan Africa (SSA), other factors contributing to sex differences in HIV epidemiology include care seeking behaviors and sociodemographic differences [11–13]. Numerous studies have found that more women than men are tested for HIV, access treatment, enroll in care earlier, and are retained in care [14–16]. According to UNAIDS, in 2020, 87% of women living with HIV knew their status, 78% of women with HIV were receiving ART, and 70% of women on ART had suppressed viral loads. During the same year, 80% of men living with HIV knew their status, 67% of men with HIV were receiving ART, and 61% of men on ART had suppressed viral loads [2]. Inequities in economic status, educational access, and gender-based violence are significant disparities that may result in unplanned treatment interruptions and may affect women more than men [17–19]. We looked to see whether these factors affected overall mortality.

The United States President's Emergency Plan for AIDS Relief (PEPFAR) Monitoring, Evaluation, and Reporting (MER) indicators were designed to monitor HIV epidemic control progress through the collection and use of programmatic disaggregated data that allow for characterization of the populations (e.g., age, sex, key and priority populations) receiving HIV services through PEPFAR support [20]. In PEPFAR-supported countries, data are routinely collected and reported to characterize the effectiveness and impact of HIV programs at the national, subnational, community, and site levels. In 2019, a new PEPFAR MER indicator, TX_ML, was introduced, enabling HIV programs to capture missed clinical visits and transfers across the treatment program, and routinely collect mortality information through programmatic data. Using TX_ML and other PEPFAR indicator data, we assessed viral load suppression (VLS) and mortality across 21 SSA countries to determine if differences existed by sex.

## Methods

We extracted data from the PEPFAR MER indicator database for persons aged 15 years and older in 21 SSA countries^1^ for the reporting period from October – December 2020 for four PEPFAR indicators (TX_CURR, TX_PVLS, TB_ART, and TX_ML) [20]. Data were collected and reported at the facility level from subnational units (SNUs) [e.g., districts, provinces, or other administrative levels].

TX_CURR captures the number of individuals currently receiving ART during the reporting period and is reported quarterly (Table 1). Individuals who are 28 days past their expected clinical contact or drug pick-up are considered to have experienced an interruption in treatment (IIT) and are not included in TX_CURR. Similarly, individuals who restart treatment after four weeks or more of being off ART are not included in TX_CURR for the reporting period. TX_PVLS presents the proportion of individuals on ART (for at least 3 months or according to national guidelines) with a documented suppressed viral load (VL) result (< 1000 copies/ml) within the past 12 months. TB_ART captures the proportion of new and relapsed TB cases in PLHIV on ART during TB treatment. The numerator for TB_ART is the number of TB cases with documented HIV-positive status who start or continue ART during the reporting period. TX_ML captures individuals on ART at the beginning of the quarterly reporting period and then had no known clinical contact since their last expected contact. TX_ML includes five outcomes: died, IIT after less than 3 months on treatment, IIT after 3 or more months on treatment, transferred out, and refused or stopped treatment. Individuals included in the “died” outcome are confirmed as dead by direct observation or by unambiguous report of family or close contact (e.g., neighbors, co-workers). Unreported deaths are not included in TX_ML; thus, it is recognized that TX_ML may underestimate deaths. The IIT categories include ART patients for whom tracing was not attempted, patients for whom tracing was attempted but was unsuccessful, and patients for whom current status cannot otherwise be determined. PEPFAR-reported data for these indicators are not de-duplicated, and thus, the indicators do not represent a cohort.

**Table 1:** Description of PEPFAR MER indicators used in the analysis

Indicator Name	Numerator	Denominator	Reporting Frequency	Notes
TX_CURR	Number of adults and children currently receiving antiretroviral therapy (ART)	N/A	Quarterly	TX_CURR restricted in the analysis to patients aged 15 years and older
TX_PVLS	Number of ART patients with suppressed viral load (VL) results (< 1,000 copies/ml) documented in the medical or laboratory records/laboratory information system (LIS) within the past 12 months	Number of ART patients with a VL result documented in the medical or laboratory records/LIS within the past 12 months	Quarterly	
TB_ART	Number of tuberculosis (TB) cases with documented HIV-positive status who start or continue antiretroviral (ART) during the reporting period	Number of registered TB cases with documented HIV-positive status during the reporting period	Quarterly	
TX_ML	Number of ART patients (currently on ART or newly initiating ART) with no clinical contact or ARV pick-up for greater than 28 days since their last expected clinical contact or ARV pick-up	N/A	Quarterly	The TX_ML indicator includes five outcomes; however, in the calculation of the upper limit mortality, only 3 disaggregate outcomes were used (died, interruption in treatment (IIT) after less than 3 months on treatment, and IIT after 3 or more months on treatment))

Viral load testing coverage was calculated for each SNU as the total number of ART patients with a VL result (from TX_PVLS, which provides numerators and denominators separately) divided by the number of individuals receiving ART 6 months prior to the reporting period (from TX_CURR). The HIV/TB coinfection rate (defined as co-occurrence of HIV infection and the disease tuberculosis) was calculated using the numerator of TB_ART (for known and new TB cases) with TX_CURR from the same quarter as the denominator. VLS-adjusted death rate per 1000 persons was calculated as the number of individuals documented as having died since last known clinical contact (from TX_ML) divided by the number of individuals receiving ART as of the reporting period (from TX_CURR) and including those documented as having died in the denominator (as they are excluded from TX_CURR for the period) multiplied by 1000 for standardization. Because many deaths are likely not documented and may instead be counted in the other disaggregate indicators for TX_ML, we performed a sensitivity analysis to determine whether the results would change if we used a much more liberal estimate of the death rate. This liberal estimate was calculated by adding the TX_ML outcomes of IIT after less than 3 months on treatment, IIT after 3 or more months on treatment, and documented as died divided by the number of individuals receiving ART as of the study period plus those documented as having died as the denominator. Refusals or stopped treatments were excluded from this calculation as, although they did experience an IIT, a final disposition was known for these individuals.

Extracted data were at the facility-level and disaggregated by sex and age group (15–49 and 50 +) for each SNU. We used SAS 9.4 to fit regression models to estimate: 1. proportion of VLS, and 2. rate of reported deaths and reported deaths plus IIT adjusting for VLS at the SNU level. Linear regression models were fit for VLS model and negative binomial models for the reported deaths and deaths plus IIT models. Using the regression models, we estimated adjusted means for males and females and for ages 15–49 and 50 + . We examined residual plots for the VLS models to ensure that SNU-level proportion residuals were roughly normally distributed, meeting the assumptions of the models. All models were weighted for SNU-level ART population size (TX_CURR) and adjusted for sex, age, HIV/TB coinfection rate, country and SNU; reported deaths and deaths plus IIT models were additionally adjusted for mean VLS at the SNU-level.

## Results

Twenty-eight of 300 SNUs (9.3%) were excluded from the analysis, one because TX_PVLS was substantially larger than TX_CURR, leading to a VLS greater than 100%, and 27 because they did not have all of the four sex x age groups represented. The mean number of individuals on ART across SNUs was 53,675 (range: 303–1,078,960). Women and individuals within the 15–49 age group constituted the majority of all individuals on ART (66.7% and 78.9%, respectively). Among those included in the TX_CURR indicator, 4.6% experienced IIT (including death) during the reporting quarter and 0.2% were documented as having died. Among those included in the TX_ML indicator, 67.7% experienced IIT after 3 or more months on treatment, 20.4% transferred to another facility, 6.5% experienced IIT after less than 3 months on treatment, 4.5% were documented as having died, and 0.8% refused or stopped treatment.

Overall mean VL testing coverage across SNUs was 79.2% and was higher among women (80.8% vs. 76.3%, *p* = 0.001) and among those in the 50 + age group (86.0% vs. 77.4%, *p* < 0.0001). Overall mean VLS was 92.5% and was slightly higher among women than men (92.8% vs. 91.9%, *p* = 0.030) and in the 50 + than the 15–49 group (94.4% vs. 91.9%, *p* < 0.0001). Among individuals on ART, mean HIV/TB coinfection rate was 0.3% (range: 0–1.8%), was higher among men than women (0.6% vs. 0.2%, *p* < 0.0001) and was similar in the 50 + age group (0.4% vs. 0.3%, *p* = 0.217). Overall mean TX_ML (treatment mortality and loss) was 4.2% and was similar among men and women (4.5% vs. 4.1%, *p* = 0.1179) and higher in the 15–49 than the 50 + group (4.5% vs. 3.2%, *p* < 0.0001).

Modeled mean VLS proportion among SNUs was higher among women than men (93.0% vs. 92.0%, *p*-value < 0.0001) and among the 50 + than 15–49 age group (93.7% vs. 91.2%, *p*-value < 0.0001) (Table 2). Controlling for SNU-level VLS, the mean death rate was higher among men as compared to women (2.37 vs. 1.51 per 1000 persons, *p*-value < 0.0001) and among those in the 50 + as compared to the 15–49 group (2.39 vs. 1.50 per 1000 persons, *p*-value < 0.0001). The rate of death plus IIT was also significantly higher among men than women (30.1 vs. 26.0 per 1000 persons, *p*-value < 0.0001). The rate of death plus IIT was higher in the 15–49 than the 50 + group (34.7 vs. 22.6 per 1000 persons, *p*-value < 0.0001).

**Table 2:** Estimates of mean viral load suppression (VLS) and VLS-adjusted deaths (including interruptions in treatment (IIT))^ab^

	n	N	Mean	β^c^	95% CI	p-value
Model 1: VLS%^d^						
Age group						< .0001
15–49	7,771,100	8,324,385	91.2		89.3, 93.2	
50 +	2,303,550	2,404,957	93.7		91.8, 95.7	
Sex						< .0001
Male	3,187,377	3,417,224	92.0		90.0, 93.9	
Female	6,887,273	7,312,118	93.0		91.0, 94.9	
HIV/TB coinfection rate (1-point increase)				-0.91		< .0001
Model 2: VLS-adjusted death rate per 1,000 persons^e^						
Age group						< .0001
15–49	18,149	11,530,619	1.50		1.05, 2.13	
50 +	8,718	3,095,920	2.39		1.68, 3.40	
Sex						< .0001
Male	12,659	4,867,450	2.37		1.67, 3.37	
Female	14,208	9,759,089	1.51		1.06, 2.15	
VLS (5-point increase)				0.93	0.87, 0.99	.0220
HIV/TB coinfection rate (1-point increase)				1.24	1.11, 1.38	< .0001
Model 3: VLS-adjusted rate of death plus IIT per 1,000 persons^e^						
Age group						< .0001
15–49	603,178	12,115,648	34.69		24.21, 49.70	
50 +	104,924	3,192,126	22.59		15.77, 32.37	
Sex						< .0001
Male	249,937	5,104,728	30.09		21.00, 43.12	
Female	458,165	10,203,046	26.04		18.17, 37.30	
HIV/TB coinfection rate (1-point increase)				1.08	1.02, 1.15	.0094
VLS (5-point increase)				1.00	0.96, 1.04	.8665

^a^Models included age group, sex, country, and HIV/TB coinfection rate

^b^All models were weighted for SNU-level ART population size and adjusted for sex, age, HIV/TB coinfection, and country; mortality models were also adjusted for SNU-level VLS

^c^For the VLS model, β is the decrease in VLS associated with a 1-point increase in the HIV/TB coinfection rate; for mortality models, β is the event rate ratio associated with a 1-point increase in HIV/TB coinfection or a 5-point increase in VLS

^d^For VLS, n refers to the number of antiretroviral therapy (ART) patients with suppressed VL results (< 1,000 copies/ml) and N refers to the total number of ART patients with a VL result documented in the medical or laboratory records/laboratory information system (LIS) within the past 12 months

^e^For VLS-adjusted deaths, n refers to the number of individuals documented dead since last known clinical contact and N refers to the number of individuals receiving ART during the reporting period, including those documented dead. For VLS-adjusted deaths plus IIT, n refers to the sum of the number of individuals who experienced IIT after less than 3 months on treatment, number who experienced IIT after 3 or more months on treatment, and number documented dead, and N refers to the number of individuals receiving ART during the reporting period

## Discussion

Our analysis found that mortality was higher among men, even after adjusting for VLS, and higher in the 50 + age group in the reported deaths model, but lower in the 50 + age group in the reported deaths plus IIT model. VLS was slightly higher among women and more women than men were reported to be on treatment. These findings suggest possible sex and age group differences in HIV treatment.

The late engagement of men in HIV prevention and treatment has long been a barrier to ending HIV as a public health threat. Among a 2019 cohort of 20 African countries, 4.7% of HIV tests conducted among men (*n* = 20.7 million) resulted positive as compared to 4.1% (*n* = 40.3 million) among women, suggesting that men are simultaneously less likely to be tested for HIV and more likely to test positive for HIV than women. Furthermore, data from adults in the African Cohort Study (AFRICOS) show that men had increased odds of advanced disease at enrollment (OR: 1.38, 1.12–1.71) [21]. A 2017 study from South Africa found that at the time of HIV diagnosis, men were more likely to already present with advanced HIV disease, which has implications for adverse outcomes [22]. Building upon these findings, a recent review examining sex differences in HIV treatment drew similar conclusions: men have a higher death rate than women in South Africa, even when accounting for differences in the timing of presentation to care; excess deaths occurred in men in Botswana with TB listed as the leading cause of death; and mortality rates were higher in males in four sub-Saharan African countries between 2005 and 2010, a finding attributed in this group to late presentation among males [23].

HIV-associated TB mortality has long been a significant public health concern in SSA. The WHO calculates a WHO TB-HIV incident coinfection rate for the African region comprised of 47 member states. The latest WHO estimate for HIV-positive TB incidence was 42 per 100,000 per year [24]. Captured in our HIV/TB coinfection estimate of 0.3% (or 300 per 100,000) are both newly identified and relapsed TB cases. Among the 18,890 known and new TB cases who initiated or continued ART during the reporting period of our analysis, more than half were relapsed TB cases, likely driving our HIV/TB coinfection rate higher.

Overall VLS among the 21 SSA countries included in our analysis (92.5%) indicates promising progress towards the UNAIDS goal of 95% of all persons receiving ART achieve viral suppression. A recent study examining scale-up of HIV viral load testing during 2013–2018 in eight SSA countries found similar results. From 2013 to 2018, the total number of patients receiving ART increased by 78% across all eight countries. Prior to scale-up activities, the rate of VLS was > 80% in only three of eight countries; however, by the end of 2018, all but one country had reported VLS rates of > 85% [25]. Treatment remains one of the most effective methods to prevent HIV transmission. Monitoring population-level viral suppression is important to understand progress towards ending HIV as a public health threat.

A 2015 systematic review and meta-analysis examining data from 106 cohorts found that the leading causes of hospital admissions and deaths among adult PLHIV worldwide were AIDS-related illnesses (46%) and bacterial infections (31%) [26]. Individuals with AHD require intensive care and experience a greater morbidity and mortality than those without advanced HIV disease. The WHO recommends a package of interventions consisting of screening, prophylaxis, and treatment of OIs to reduce mortality from AHD [27–29]. Data on advanced HIV disease are not explicitly collected through PEPFAR; however, data from the Population-Based HIV Impact Assessment (PHIA) Project and from AFRICOS suggest that the proportion of individuals presenting with advanced disease at diagnosis is as high as 20% in many countries [21, 30, 31]. Since mortality estimates often underrepresent true burden, the incorporation of the other TX_ML disaggregates into the upper limit mortality calculation provides a wide range in which a more accurate mortality estimate may fall. Coupled with data on viral suppression, our findings provide additional signals on areas of improvement in linkage to care and treatment retention. Among the 21 SSA countries included in our analysis, VLS was slightly higher among women, and reported deaths were 1.5 times higher among men despite adjusting for VLS. Efforts to find and engage men in HIV testing and initiate and retain them in treatment should be considered and may result in improved outcomes throughout the care continuum. It remains to be seen whether overall increased rates of tuberculosis are driving the higher death rates in men compared to women.

There are limitations to the interpretation of our findings. First, MER indicator data are cross-sectional, aggregate, and unlinked and, as such, may include duplicate data. In addition, MER data overall are subject to at least two major limitations: a) analysis across multiple indicators is difficult, as each indicator captures a distinct group of patients and cannot be linked in the way that case surveillance data allows; and b) although MER reporting occurs quarterly, the structure of the data precludes analysis of certain indicators, namely TX_ML, across time periods. This is because TX_ML estimates may fluctuate from one reporting period to the next, as individuals may move across outcome disaggregates and/or be re-engaged in care and no longer experiencing IIT. Therefore, this analysis was limited to one quarterly reporting period. Secondly, TX_ML data likely underestimate the true burden of mortality. TX_ML only captures mortality among ART recipients who experience IIT, thus failing to capture mortality data on PLHIV not currently receiving treatment. Among those reported as having died, cause of death (COD) information is largely missing or unreliable as COD are not mandatorily reported within TX_ML. Furthermore, HIV programs attempt to locate and re-engage in care patients that have experienced IIT; however, if a patient is unable to be traced, it remains unknown whether that patient was truly lost to follow-up, transferred to another facility without documentation, or died. Having included data on age in two age groups, rather than in finer age disaggregates, may also have limited our ability to observe nuanced differences across age groups. Notably, the COVID-19 pandemic, which posed significant challenges to routine HIV care globally, may have impacted our study, given the timing of data collection. Innovations were introduced into the care continuum (i.e., differentiated service delivery models) to ensure continuity of HIV services through PEPFAR programs; however, it is possible that treatment and follow-up may have been impacted. Lastly, our findings are likely confounded by other factors not included in this analysis, including time since diagnosis, presence of comorbidities, health-seeking behaviors during a pandemic, and biological, sociodemographic, behavioral differences between the sexes, and our ability to statistically control for individual-level factors is limited when using aggregate facility-level data. While these are significant limitations, each of them—measurement error, conduct of the analysis at the SNU rather than individual level, and incomplete reporting—should bias the findings in the direction of the null hypothesis rather than in the direction of strong relationships between variables.

With the above limitations acknowledged, the main strength of this analysis is the use of routinely collected data from a large number of countries to assess sex differences in HIV treatment outcomes. TX_ML is the only source of mortality data on PLHIV on a national level for most countries included in this analysis. Through the routine reporting of continuity of treatment and mortality, TX_ML helps us to better understand reasons for missed clinical visits, encourages tracing of PLHIV when they have had no clinical contact, and promotes timely identification of patient outcomes among PLHIV known to have missed clinical visits or medication pickups. In countries where HIV case surveillance (CS) is operational, however, demographic and health event data among PLHIV are systematically and continuously collected from time of diagnosis to time of death, allowing for better characterization of the HIV epidemic and improvement of HIV programs. In the absence of, or as a supplement to CS, timely and routine review and analysis of TX_ML and other PEPFAR MER indicator data can inform programmatic development which has the potential to both add to the scientific inquiry and achieve better outcomes at the public health level.

## Conclusions

Results from this study lend further scientific evidence that sex and age group differences may persist in HIV treatment outcomes in SSA countries and supports the notion that men have higher mortality, a finding likely driven by late access to care, poor engagement, advanced HIV disease, and potentially tuberculosis. Mortality data may be used to target and deploy the advanced HIV disease package of care in the SNUs with the highest mortality burden. Additionally, these data may be used to inform and advocate for policy changes to include more extensive testing for identification of AHD. In SSA, greater attention should be placed on the prevention, testing, and treatment of men to lessen the divide between the sexes and continue the progress towards epidemic control. Additionally, in countries with a high HIV burden, establishment of routine mortality surveillance (through various methods including civil registration and vital statistics, verbal autopsy, and mortuary surveillance) may assist HIV programs in understanding the impact of treatment programs. Future studies using more robust mortality data stratified by country and integrating OIs and non-communicable diseases into treatment models may be conducted to further investigate the relationship between biological sex, AHD, and mortality.

## Data Availability

Some of the PEPFAR MER data analyzed in this work are made publicly available through the PEPFAR Panorama Spotlight site (PEPFAR Panorama Spotlight). Remaining data are available from the corresponding author on reasonable request.

## Abbreviations

AFRICOS: African Cohort Study
AHD: Advanced HIV disease
ART: Antiretroviral therapy
COD: Cause of death
CS: HIV case surveillance
IIT: Interruption in treatment
MER: Monitoring, Evaluation, and Reporting
OI: Opportunistic infection
PEPFAR: United States President’s Emergency Plan for AIDS Relief
PHIA: Population-Based HIV Impact Assessment
PLHIV: People living with HIV
SNU: Subnational unit
SSA: Sub-Saharan Africa
UNAIDS: Joint United Nations Programme on HIV/AIDS
VL: Viral load
VLS: Viral load suppression

## Footnotes

1 Angola, Botswana, Burundi, Cameroon, Cote d’Ivoire, Democratic Republic of Congo, Eswatini, Ethiopia, Kenya, Lesotho, Malawi, Mozambique, Namibia, Nigeria, Rwanda, South Africa, South Sudan, Tanzania, Uganda, Zambia, and Zimbabwe.
